# Development of a Chinese‐Specific Clinical Model to Predict Maturity‐Onset Diabetes of the Young

**DOI:** 10.1002/dmrr.70087

**Published:** 2025-09-18

**Authors:** Sandra T. F. Tsoi, Cadmon K. P. Lim, Ronald C. W. Ma, Eric S. H. Lau, Baoqi Fan, Chun Kwan O, Yingnan Fan, Elaine Chow, Alice P. S. Kong, Wing‐Yee So, Juliana C. N. Chan, Andrea O. Y. Luk

**Affiliations:** ^1^ Department of Medicine and Therapeutics The Chinese University of Hong Kong Prince of Wales Hospital Hong Kong Hong Kong; ^2^ Hong Kong Institute of Diabetes and Obesity The Chinese University of Hong Kong Prince of Wales Hospital Hong Kong Hong Kong; ^3^ Li Ka Shing Institute of Health Sciences The Chinese University of Hong Kong Prince of Wales Hospital Hong Kong Hong Kong

**Keywords:** Chinese, maturity‐onset diabetes of the young (MODY), monogenic diabetes, prediction models

## Abstract

**Aims:**

Accurate identification of individuals with maturity‐onset diabetes of the young (MODY) can support precision diabetes management. However, diagnosing MODY is challenging due to overlapping clinical features with type 2 diabetes. We aimed to develop a prediction model for identifying Chinese with high likelihood of MODY for further genetic testing.

**Methods:**

We developed a logistic regression model using clinical data from an unselected cohort of 1021 Chinese with young‐onset (age at diagnosis ≤ 40) non‐type 1 diabetes enrolled in the Hong Kong Diabetes Register, 1.9% (*n* = 19) of whom had MODY (*GCK*‐, *HNF1A*‐, *HNF4A*‐ and *HNF1B*‐MODY) by molecular confirmation. We validated the model in an independent local cohort of 822 Chinese with young‐onset non‐type 1 diabetes. We compared the performance of the new Chinese‐specific MODY prediction model with an existing MODY probability calculator in the validation cohort.

**Results:**

The prediction model comprised the following clinical variables: current age, age at diagnosis, sex, body mass index, systolic blood pressure, HDL‐cholesterol, LDL‐cholesterol, triglyceride and fasting C‐peptide. It demonstrated acceptable discrimination of patients with MODY in the validation dataset, with an area under the curve of 0.813 (95% confidence interval 0.647–0.979). At the probability cut‐off of 50%, the model achieved a sensitivity of 72.7% and a specificity of 92.4%. It allows identification of one MODY case in every nine genetic tests conducted.

**Conclusion:**

We developed a comprehensive Chinese‐specific MODY prediction model. This model can be used in unselected Chinese with young‐onset non‐type 1 diabetes to identify high‐risk individuals for genetic testing.

**Abbreviations**
AUC: Area under the curve
MODY: Maturity‐onset diabetes of the young
NGS: Next‐generation sequencing
PPV: Positive predictive value

## 1 Introduction

Monogenic diabetes accounts for 2%–3% of diabetes cases in Chinese individuals with young‐onset diabetes presenting at or before the age of 40. Of these cases, 80% are contributed by variants in common genes associated with maturity‐onset diabetes of the young (MODY), including glucokinase (*GCK)*, hepatic nuclear factor (*HNF)‐1A*, *HNF1B* and *HNF4A* [1]. A correct diagnosis of MODY can guide treatment, inform disease prognosis, and facilitate cascade screening for family members [2, 3]. For instance, individuals with *GCK*‐MODY generally do not require glucose‐lowering drugs, as their hyperglycemia is mild and their risk of diabetes‐related complications is low [2]. In contrast, individuals with *HNF1A*‐ and *HNF4A*‐MODY have significant post‐meal glucose excursions, which are sensitive to the action of sulfonylureas; therefore, this class of drugs should be used preferentially for those affected [4].

A diagnosis of MODY can only be confirmed through genetic testing. Early guidelines recommended genetic testing for individuals diagnosed with diabetes before the age of 25, those with a family history of diabetes, and those who are non‐insulin dependent [5]. However, such criteria had low differentiating value in identifying individuals with a high probability of MODY for genetic testing, resulting in under‐diagnosis of the condition in most clinical settings. This challenge is further exacerbated by the low level of disease awareness and the high costs associated with sequencing [6]. Biomarkers such as high‐sensitivity C‐reactive protein and 1,5‐anhydroglucitol, used alone or in combination with other clinical parameters, have shown reasonable accuracy in identifying individuals with *HNF1A*‐MODY [7, 8, 9, 10]. Another risk score that incorporates clinical and radiographic features has been shown to predict *HNF1B*‐MODY [11]. Nevertheless, the usability of these algorithms is hampered by the requirement for non‐routine biomarkers and imaging studies. In 2012, a MODY probability calculator was developed using a weighted combination of clinical criteria in a population of white Europeans with MODY, type 1 diabetes, or type 2 diabetes to discriminate cases of MODY from other diabetes subtypes [12]. Given the differences in phenotypes across ethnic groups, it is not known whether the MODY probability calculator constructed for white Europeans is applicable to Chinese in whom young age at diabetes presentation, lean body habitus, and a strong family history of diabetes are common features [13, 14, 15].

Young‐onset diabetes is prevalent in the Chinese population, accounting for up to one in five individuals with diabetes [16]. Only 6% of diabetes cases presenting at or before the age of 40 are classified as type 1 diabetes, while the remaining cases are categorised as non‐type 1 diabetes, typically type 2 diabetes. However, a subset of these individuals, who are considered to have type 2 diabetes, may carry genetic variants for MODY or may have latent autoimmune diabetes [17]. The aims of this study are twofold: (1) to develop and validate a Chinese‐specific prediction algorithm to estimate the probability of MODY, and (2) to compare the performance of this Chinese‐specific prediction algorithm with an existing MODY probability calculator in Chinese with young‐onset non‐type 1 diabetes.

## 2 Methods and Study Cohorts

### 2.1 Study Cohorts

The Hong Kong Diabetes Register (HKDR), established in 1995 as part of a quality improvement programme, enrolled Chinese individuals with physician‐diagnosed diabetes who were referred to the Diabetes and Endocrine Centre at the Prince of Wales Hospital, Hong Kong Special Administrative Region, for a structured assessment of metabolic control and diabetes complications [18, 19]. Referral sources included hospital‐based specialist out‐patient clinics, family medicine out‐patient clinics, and community‐based out‐patient clinics. Between 1995 and 2012, 21,366 patients were enrolled. A subset of these enrollees consented to the donation and archival of additional blood specimens for future biomedical research. In an earlier study, we performed next‐generation sequencing (NGS) of genes related to monogenic diabetes, including common MODY genes such as *GCK*, *HNF1A*, *HNF1B*, and *HNF4A*, in 1021 individuals with non‐type 1 diabetes diagnosed at or before the age of 40 in the cohort [1]. Type 1 diabetes was defined based on either the presentation of diabetic ketoacidosis and/or the requirement of insulin therapy within 12 months of diagnosis. These 1021 patients were included as the training dataset for the development of the prediction algorithm.

The Precision Medicine to Redefine Insulin Secretion and Monogenic Diabetes (PRISM) study was a 3‐year randomised controlled trial (RCT) designed to evaluate the effects of precision treatment guided by biogenetic markers in Chinese individuals with young‐onset diabetes [20]. Between 2020 and 2021, 884 participants diagnosed with non‐type 1 diabetes at or before the age of 40 were enrolled. Comprehensive biogenetic profiling, including targeted sequencing for monogenic diabetes, was performed [1]. After excluding individuals who were also enrolled in the HKDR, 822 participants from the PRISM cohort were included in this study as the validation dataset.

All individuals provided written informed consent, indicating their agreement to contribute their anonymised clinical data for research.

### 2.2 Genetic Testing

Targeted sequencing of genes associated with monogenic diabetes as well as analysis of copy number variation (CNV) in the *HNF1B* gene were performed as described in our previous study (Supporting Information S1: Table S1) [1]. An individual was considered to have MODY if he or she carried pathogenic or likely pathogenic variants in *GCK*, *HNF1A*, *HNF1B*, and/or *HNF4A* genes (Supporting Information S1).

### 2.3 Model Training and Validation

We used logistic regression and random forest, respectively, to develop prediction models in the training dataset (Supporting Information S1: Figure S1). The clinical variables for model building were selected based on statistically significant differences in values between individuals with and without MODY. Details on variable selection methods and the justification for the final set of variables used are described in Supporting Information S1.

Logistic regression was used to predict the probability of having MODY [21]. To mitigate the risk of overfitting associated with limited sample size, we applied Ridge regularisation and adjusted for class imbalance during model development. The clinical variables were tested for multicollinearity, and only those with a variance inflation factor (VIF) below 5 were retained in the prediction model construction. The intercept and regression coefficients of each independent variable were determined in the training dataset. Odds ratio (ORs) for each variable were calculated by exponentiating the regression coefficients, and the corresponding 95% confidence intervals (CIs), standard errors, *z* scores, and *p*‐values were obtained from a generalised linear model (GLM) with a logit link function. Additionally, we performed bootstrap resampling with 10,000 iterations to derive empirical estimates of the 95% CIs, standard errors, *z* scores, and *p*‐values to evaluate model variability under a limited number of events.

Random forest, which consists of multiple decision trees formed by randomly selected variables, was the second approach employed for model development [22]. The parameters of the random forest, such as the number of trees and layers, were optimised by hyperparameter tuning with cross‐validation in the training dataset. Using a balanced random forest classifier, a probability was predicted for each individual based on their clinical data, indicating the proportion of trees in the forest that voted for a particular class [22].

The models were validated using the PRISM cohort (Supporting Information S1: Figure S1). Missing data for specific variables among participants were imputed by the median value of the variable from the corresponding dataset (Supporting Information S1: Table S2).

### 2.4 Existing MODY Probability Calculator

We compared the performance of the Chinese‐specific MODY prediction model against an existing MODY probability calculator developed by Shields and colleagues at the University of Exeter, United Kingdom, which differentiates MODY from type 2 diabetes [12]. The Exeter MODY probability calculator was constructed using a logistic regression model that incorporates the following clinical parameters: current age, age at diabetes diagnosis, sex, body mass index (BMI), HbA1c value, parental history of diabetes, current glucose‐lowering drug therapy (yes or no), and time from diabetes diagnosis to insulin therapy. Of note, the Exeter MODY probability calculator is applicable only to individuals diagnosed with diabetes before the age of 35.

### 2.5 Statistical Analysis

Continuous variables are presented as medians (interquartile range [IQR]) for variables with a skewed distribution. Categorical variables are presented as numbers (%). The Chi‐squared test or Fisher's Exact test was used for the comparison of categorical variables, and the Wilcoxon rank‐sum test was used for the comparison of nonparametric data when appropriate. Results were considered to be statistically significant when the two‐sided *p*‐value was less than 0.05.

The optimal threshold of each model was determined by identifying the highest Youden's J statistic, calculated as ‘sensitivity + specificity −1’ by cross‐validation in the training dataset. Receiver operating characteristics (ROC) curves and the area under the curve (AUC) were used to measure the discriminative ability of the models in both the training and validation datasets [21]. Performance metrics, including sensitivity, specificity, positive predictive value (PPV), and false negative rate (FNR), were used to evaluate the performance of the newly constructed prediction models in the validation dataset. All statistical tests were performed using Python (version 3.9.12, Python Software Foundation), supported by multiple packages, as well as the Statistical Package for Social Science (SPSS) (version 26.0, IBM Corporation).

## 3 Results

### 3.1 Genetic Results

Among 1021 Chinese individuals with non‐type 1 diabetes in the training dataset, 19 (1.9%) individuals carried pathogenic or likely pathogenic variants in *GCK* (*n* = 6), *HNF1A* (*n* = 9), *HNF1B* (*n* = 3), or *HNF4A* (*n* = 1) genes. Among 822 Chinese with non‐type 1 diabetes in the validation dataset, causative variants were detected in 11 (1.3%) individuals (Supporting Information S1: Figure S2).

### 3.2 Clinical Characteristics

In the training cohort, individuals with confirmed MODY were younger at the age of diabetes diagnosis compared with those who tested negative for causative genetic variants in MODY genes (31.0 [19.0–35.0] vs. 35.0 [30.0–38.0] years, *p* = 0.017). A greater proportion of individuals with MODY were diagnosed with diabetes before the age of 25 (26.3% vs. 9.7%, *p* = 0.034), without significant difference in sex distribution (Supporting Information S1: Table S3). Individuals with MODY had more favourable metabolic indices, including a lower BMI (21.6 [20.1–23.8] vs. 25.5 [22.7–28.8] kg/m^2^, *p* < 0.001), lower waist circumference (71.8 [71.0–93.0] vs. 89.0 [81.0–96.0] cm for men, *p* = 0.047; 70.0 [65.3–77.5] vs. 83.0 [76.0–90.0] cm for women, *p* < 0.001), decreased systolic blood pressure (116.0 [109.5–123.5] vs. 123.8 [114.0–136.0] mmHg, *p* = 0.038), decreased low‐density lipoprotein (LDL)‐cholesterol (2.40 [1.97–2.90] vs. 2.83 [2.30–3.50] mmol/L, *p* = 0.038), decreased triglyceride (0.88 [0.70–1.28] vs. 1.38 [0.90–2.17] mmol/L, *p* = 0.005), and increased high‐density lipoprotein (HDL)‐cholesterol (1.60 [1.08–1.87] vs. 1.24 [1.01–1.50] mmol/L, *p* = 0.007). Glycaemic indices, including HbA1c and fasting plasma glucose levels, were similar between the two groups, but individuals with MODY had lower C‐peptide levels (50.0 [50.0–223.1] vs. 265.5 [50.0–625.2] pmol/L, *p* = 0.005). The frequencies of micro‐ and macrovascular complications, as well as the use of insulin, non‐insulin glucose‐lowering drugs, and lipid‐lowering drugs, were similar across both groups. Clinical characteristics did not differ between individuals with *GCK*‐MODY and those with MODY related to transcription factors (*HNF1A*, *HNF1B* and *HNF4A*) (Supporting Information S1: Table S4). The clinical characteristics of individuals with MODY were largely similar between those enrolled in the HKDR and in the PRISM study, with the exception of a longer diabetes duration, higher fasting C‐peptide levels, and more frequent use of lipid‐lowering drugs among individuals from the PRISM cohort (Supporting Information S1: Table S5).

### 3.3 Logistic Regression and Random Forest Models Developed in Training Dataset

After testing for multicollinearity, the logistic regression model included the following nine variables: sex, current age, age at diagnosis, BMI, systolic blood pressure, HDL‐cholesterol, LDL‐cholesterol, triglyceride, and fasting C‐peptide. Waist circumference was linearly correlated with BMI and was unselected in the model. The variable coefficients of the logistic regression model along with *p*‐values derived from a GLM are presented in Table 1. Although the bootstrap‐derived CIs were generally wider than those from GLM, they remained consistent in the direction of associations and supported the overall model structure under a limited number of events (Supporting Information S1: Table S6). The random forest model, not constrained by multicollinearity, retained waist circumference as a significant variable. Examples of trees from the random forest model are shown in Supporting Information S1: Figure S3.

**TABLE 1:** Logistic regression model coefficients for MODY prediction.

Variable	β	OR	95% CI OR	SE	z score	*p*‐value
Sex (male = 0, female = 1)	0.325	1.384	0.977–1.986	0.18	1.83	0.067
Current age (years)	−0.058	0.943	0.922–0.965	0.01	−5.05	< 0.001***
Age at diagnosis (years)	−0.021	0.979	0.948–1.012	0.02	−1.23	0.221
BMI (kg/m^2^)	−0.171	0.843	0.803–0.887	0.03	−6.69	< 0.001***
Systolic blood pressure (mmHg)	0.014	1.014	1.003–1.024	0.01	2.51	0.012*
HDL‐cholesterol (mmol/L)	0.634	1.885	1.170–3.317	0.27	2.55	0.011*
LDL‐cholesterol (mmol/L)	−0.352	0.703	0.572–0.859	0.10	−3.43	0.001**
Fasting C‐peptide (pmol/L)	−0.005	0.995	0.994–0.996	0.00	−8.88	< 0.001***
Triglyceride (mmol/L)	−0.161	0.851	0.633–1.164	0.16	−0.98	0.325

*Note: β* coefficients and odds ratios (ORs) were obtained from Ridge‐regularised logistic regression, while 95% confidence intervals (CIs), standard errors (SEs), *z* scores and *p*‐values were derived from a generalised linear model (GLM).

Abbreviations: β, regression coefficients; CI, confidence interval; OR, odds ratio; SE, standard error.

*p < 0.05, **p < 0.01, ***p < 0.001.

### 3.4 Validation in PRISM Cohort

**FIGURE 1:**
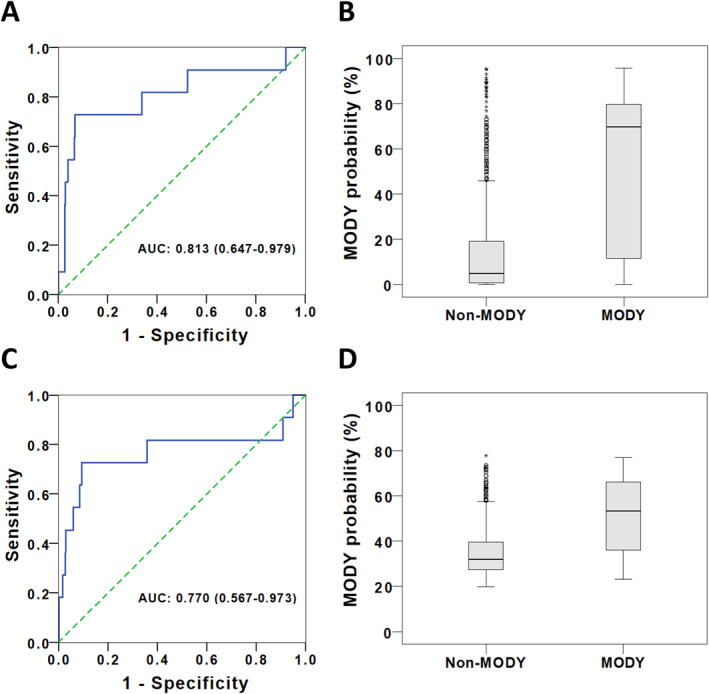
Performance of the Chinese‐specific prediction models in validation dataset: Receiver operating characteristics curves and predicted MODY probability in individuals with MODY and without MODY by logistic regression model (A, B) and random forest model (C, D).

The developed prediction algorithms were validated in the PRISM cohort. In the ROC analysis, both the logistic regression and random forest models showed acceptable discriminatory ability for MODY, with an AUC of 0.813 (95% CI 0.647–0.979) for the logistic regression model and 0.770 (95% CI 0.567–0.973) for the random forest model (Figure 1). Using the logistic regression model, individuals without MODY had a significantly lower predicted probability as compared to those with genetically confirmed MODY (0.049 [0.008–0.191] vs. 0.697 [0.116–0.797], *p* < 0.001) (Figure 1B). Similarly, the random forest model predicted a lower probability of MODY in individuals without the condition, although the difference between the two groups was smaller (0.320 [0.274–0.396] vs. 0.533 [0.360–0.661], *p* = 0.002) (Figure 1D).

At the optimal probability threshold obtained by cross‐validation, further genetic testing was recommended for predicted probability of ≥ 54.8% in the logistic regression model and ≥ 56.2% in the random forest model. Using the logistic regression model, 6 (54.5%) out of 11 individuals with pathogenic or likely pathogenic variants in MODY genes, and 52 (6.4%) out of 811 individuals without MODY would be recommended for genetic testing at the threshold of 54.8%. At this cut‐off, the logistic regression model had a sensitivity of 54.5%, a specificity of 93.7% with a positive predictive value of 10.5%, and a false negative rate of 45.5%. Using the random forest model, the optimal threshold of 56.2% would trigger genetic testing in 5 (45.5%) out of 11 individuals with MODY and 36 (4.4%) out of 811 individuals without MODY. At this cut‐off, the random forest model had a sensitivity of 45.5%, a specificity of 95.6% with a positive predictive value of 12.2% and a false negative rate of 54.5%. In the validation cohort of unselected Chinese individuals with non‐type 1 young‐onset diabetes, the logistic regression model would identify a positive case of MODY in every 10 genetic tests, and the random forest model would identify a positive case in every eight genetic tests. Model performance at various probability cut‐offs is shown in Table 2.

**TABLE 2:** Performance of models at different probability cut‐offs in the validation dataset.

Probability cut‐off	≥ 10%	≥ 20%	≥ 30%	≥ 40%	≥ 50%	≥ 60%	≥ 70%	≥ 80%	≥ 90%
Logistic regression model^a^									
Sensitivity	81.8%	72.7%	72.7%	72.7%	72.7%	54.5%	45.5%	18.2%	9.1%
Specificity	63.5%	76.1%	83.0%	88.2%	92.4%	94.5%	96.4%	97.4%	99.4%
Positive predictive value (PPV)	3.0%	4.0%	5.5%	7.7%	11.4%	11.8%	14.7%	8.7%	16.7%
False negative rate (FNR)	18.2%	27.3%	27.3%	27.3%	27.3%	45.5%	54.5%	81.8%	90.9%
In population of 1000 people									
Number of tests needed	371.0	245.7	177.6	126.5	85.2	62.0	41.4	28.0	7.3
Number of positive cases identified/missed	10.9/2.4	9.7/3.6	9.7/3.6	9.7/3.6	9.7/3.6	7.3/6.1	6.1/7.3	2.4/10.9	1.2/12.2
Random forest model^b^									
Sensitivity	100.0%	100.0%	81.8%	72.7%	54.5%	45.5%	18.2%	0.0%	0.0%
Specificity	0.0%	0.1%	39.0%	75.7%	91.9%	96.5%	99.0%	100.0%	100.0%
Positive predictive value (PPV)	1.3%	1.3%	1.8%	3.9%	8.3%	15.2%	20.0%	NA	NA
False negative rate (FNR)	0.0%	0.0%	18.2%	27.3%	45.5%	54.5%	81.8%	100.0%	100.0%
In population of 1000 people									
Number of tests needed	1000.0	998.8	613.1	249.4	87.6	40.1	12.2	0.0	0.0
Number of positive cases identified/missed	13.4/0	13.4/0	10.9/2.4	9.7/3.6	7.3/6.1	6.1/7.3	2.4/10.9	0/13.4	0/13.4

^a^ Sex, current age, age at diagnosis, BMI, systolic blood pressure, HDL‐cholesterol, LDL‐cholesterol, triglyceride and fasting C‐peptide values were included in the model.

^b^ Sex, current age, age at diagnosis, BMI, waist circumference, systolic blood pressure, HDL‐cholesterol, LDL‐cholesterol, triglyceride and fasting C‐peptide values were included in the model.

### 3.5 Sensitivity Analysis Comparing Nine‐Variable Logistic Regression Model and Reduced Models

As multiple clinical variables were included in our prediction model, we performed a sensitivity analysis to evaluate their contribution in prediction (Supporting Information S1: Table S7). We compared the full nine‐variable model with two reduced models: one including only fasting C‐peptide and BMI, and another including only variables that were statistically significant in the full logistic regression model (Supporting Information S1 and Table 1). The reduced models showed inferior predictive performance with lower PPV (11.4% vs. 8.2% vs. 10.8%) and higher FNR (27.3% vs. 36.4%).

### 3.6 Performance of an Existing MODY Probability Calculator

We evaluated the performance of the Exeter MODY probability calculator in the PRISM cohort. Approximately half of the participants (409 out of 822) were excluded from this analysis due to various factors, including age at diabetes diagnosis above 35 (*n* = 352), missing necessary information on family history (*n* = 11), and incomplete information on glucose‐lowering drug use (*n* = 46). The area under the ROC curve was 0.681 (Supporting Information S1: Figure S4). At the suggested cut‐off of ≥ 25%, the Exeter MODY probability calculator yielded a sensitivity of 66.7% and a specificity of 64.4%, with a positive predictive value of 2.7% and a false negative rate of 33.3%.

## 4 Discussion

In this study, we developed Chinese‐specific prediction models for common MODY subtypes using logistic regression and random forest approaches. The logistic regression model demonstrated superior discriminatory ability in identifying patients with MODY among unselected Chinese individuals with young‐onset non‐type 1 diabetes. We recommend performing genetic screening at a probability cut‐off of 50% using the logistic regression model. This cut‐off is lower than the probability threshold of 54.8% based on Youden's J statistic from cross‐validation, but it yields a higher sensitivity of 72.7% at the expense of a slightly lower specificity (Table 2). This 50% cut‐off would trigger genetic screening in 9% of individuals with non‐type 1 diabetes and pick up approximately three‐quarters of MODY cases. Both Chinese‐specific prediction models performed better than the earlier MODY probability calculator developed by the Exeter research group in differentiating MODY from type 2 diabetes in the Chinese population.

### 4.1 Comparison With an Existing MODY Probability Calculator

The Exeter MODY probability calculator was originally developed for white Europeans. However, inter‐ethnic differences in the clinical phenotypes of people with diabetes may affect calculator's performance in other ethnic groups. Previous studies examining diabetes clusters using age at diagnosis, BMI, and C‐peptide showed that Chinese (41%) and Indian populations (27%) had higher proportions of individuals with non‐autoimmune severe insulin‐deficient diabetes compared to the white European population (17.5%) [23]. In our current cohort of individuals with young‐onset non‐type 1 diabetes, the median BMI was 25 kg/m^2^, with up to 30% classified as not overweight, and 60% reported a parental history of diabetes. It was not surprizing that the previous MODY probability calculator performed less effectively in Chinese individuals compared with white Europeans, likely due to inter‐ethnic differences in clinical attributes that discriminate MODY from type 2 diabetes. Likewise, an earlier study involving 1911 unselected Chinese individuals with type 2 diabetes aged 15–35, who were sequenced for 14 MODY genes, examined the performance of the Exeter MODY probability calculator [24]. Using the recommended probability cut‐off of ≥ 25%, they found that the calculator achieved a sensitivity of 60%, specificity of 73%, positive predictive value of 5%, and a missing rate of 40%, which is consistent with the results observed in our study.

There are several differences between the Exeter MODY probability calculator and our Chinese‐specific MODY prediction models. First, the previous MODY probability calculator is applicable only to individuals diagnosed with diabetes at or before the age of 35, whereas it is common for people with MODY to present with hyperglycaemia later in life. In our Chinese cohort, 20% of individuals with MODY were diagnosed between the ages of 35 and 40. This may be due to incomplete penetrance of genetic variants as have been shown for *HNF1A*, *HNF4A* and *HNF1B* [25, 26, 27, 28]. Our prediction models were developed for Chinese individuals with non‐type 1 diabetes presenting at or before the age of 40, thereby broadening the application age range beyond that of the previous MODY probability calculator. Second, our model predicted the probability of four MODY subtypes, including *GCK*‐, *HNF1A*‐, *HNF4A*‐, and *HNF1B*‐MODY. In contrast, the previous calculator did not consider *HNF1B*‐MODY in its prediction. We included *HNF1B*‐MODY because variants in *HNF1B* accounted for approximately 15% of all MODY cases in our Chinese cohort. Third, the range of clinical parameters included in the various models differed. Our Chinese‐specific prediction models incorporated lipid indices and fasting C‐peptide, while variables such as family history of diabetes, HbA1c, use of glucose‐lowering drugs or insulin, and time to insulin therapy were excluded. These latter variables did not show significant differences between individuals with and without MODY in our Chinese cohort. A family history of diabetes is common, reported in over half of Chinese individuals with young‐onset type 2 diabetes. Notably, in our study, a positive family history was operationally defined as the presence of diabetes in at least one first‐degree relative. The absence of a comprehensive evaluation of the family pedigree across multiple generations may diminish the predictive value associated with a positive family history in our study. The use of insulin and the time from diagnosis to insulin therapy are treatment‐related factors that could be influenced by physician practices, patient preferences, as well as available drug options and clinical guidelines, which may change over time. Therefore, insulin therapy may not consistently reflect the underlying disease subtype, limiting its utility in differentiating MODY from type 2 diabetes.

### 4.2 Applications of Prediction Model in Chinese Population

In a previous population‐based study examining the characteristics of Hong Kong Chinese individuals with diabetes by age categories, approximately 37,000 residents were identified with young‐onset ‘type 2 diabetes’, presenting at age 40 or below during the period from 2000 to 2018 [29]. In China, the crude prevalence of ‘type 2 diabetes’ among the youth population aged 3 to 18 was 0.18%, while the weighted prevalence in adults aged 18 to 40 was 5.9% [30, 31]. Given that close to half of China's 1.4 billion residents are under the age of 40, the number of individuals with diabetes, including MODY, in this age category is substantial. Timely diagnosis of MODY can facilitate personalisation of disease management tailored to the specific genetic variant that an individual carries. However, efficient case finding is partly limited by the overlapping clinical features of MODY and type 2 diabetes. In the PRISM cohort, none of the participants with MODY had been previously diagnosed by their usual care physicians. Our newly developed Chinese‐specific prediction model can estimate the pre‐test probability of common MODY subtypes, allowing for the selection of high‐risk individuals to undergo genetic testing. This tool will improve test efficiency, support precision treatment, and aid in prognostication and screening of at‐risk family members [2]. As it requires only routinely collected clinical variables, including BMI, blood pressure, lipid indices and fasting C‐peptide, this model can be readily adopted in clinical practice.

### 4.3 Limitations

We acknowledge the following limitations of this study. First, the algorithm is not intended to predict the probability of other less common monogenic diabetes subtypes, including neonatal diabetes, rare syndromic diabetes, and diabetes with severe insulin resistance. Insulin resistance is a common feature of type 2 diabetes, often associated with obesity, hypertriglyceridaemia, and elevated C‐peptide levels, making it challenging to differentiate between type 2 diabetes and monogenic forms of insulin resistance [32, 33, 34]. Our prediction model focuses on detecting common MODY subtypes (*GCK*‐, *HNF1A*‐, *HNF4A*‐ and *HNF1B*‐MODY), which account for over 80% of monogenic diabetes reported in Chinese [1]. Due to the limited number of cases in our cohorts, we did not evaluate the models for distinguishing between individual MODY subtype and type 2 diabetes. Second, it is possible that some individuals have MODY coexisting with obesity and its related comorbidities, including type 2 diabetes, who may not be identified in our prediction model [35, 36]. Among the 11 patients with genetically confirmed MODY in the external validation cohort, eight had a predicted probability above 50%, while the remaining three had distinctly lower probabilities of 0.1%, 4.5%, and 11.6%, respectively. These three individuals had *HNF1A*, *HNF1B*, and *GCK* variants, respectively, and they exhibited phenotypes associated with insulin resistance, including high BMI, hypertension, dyslipidaemia, and elevated fasting C‐peptide levels. Therefore, the MODY prediction model should not be viewed as a substitute for clinical expertise. Maintaining a heightened level of suspicion is necessary for detecting irregularities in clinical presentation and accurately establishing diagnoses. Third, we did not develop a separate prediction model to differentiate MODY from type 1 diabetes. The incidence of type 1 diabetes, defined by diabetic ketoacidosis at presentation or the requirement of insulin therapy within 12 months of diagnosis, is low in Chinese youth, with rates reported at five to six per 100,000 person‐year in a territory‐wide cohort in Hong Kong [17]. In contrast to white Europeans, where type 1 diabetes accounted for 85% of cases of youth‐onset diabetes (ages < 20), it comprises only 40% of all cases of diabetes presenting in youth (ages < 20) and 6% of cases presenting by age of 40 in Chinese [37, 38]. Although it is possible that a small proportion of individuals with common MODY could be misdiagnosed as having type 1 diabetes, the likelihood is low, as the clinical manifestations of type 1 diabetes are usually more recognisable. We did not exclude individuals with positive anti‐glutamic acid decarboxylase antibodies among those with non‐type 1 diabetes, as latent autoimmune diabetes and MODY can co‐exist [1]. Fourth, we acknowledge that the prediction models are significantly influenced by subtype distribution and clinical features of individuals in the training dataset. Missing information was imputed by the median of each variable, which might not accurately represent the actual characteristics of the patients. However, the percentage of data missing was low, with the highest percentage noted for LDL‐cholesterol, where 5.8% of data in the HKDR cohort and 4.7% of data in the PRISM cohort were unavailable. Finally, our model was developed in Chinese and may not be applicable to other East Asian populations. Validation in other cohorts is required to refine the proposed probability threshold for genetic testing.

In summary, we developed a prediction model for MODY in Chinese individuals presenting with non‐type 1 diabetes at or before 40 years of age, aimed at supporting decision making on genetic testing. The model was validated in an independent local cohort and showed promising results, identifying three‐quarters of individuals with MODY while yielding a positive case in every nine individuals tested among unselected Chinese patients with young‐onset non‐type 1 diabetes. The application of this prediction model in a clinical setting to determine the pre‐test probability of MODY may enhance test efficiency and facilitate precision management of young‐onset diabetes in the Chinese population.

## Author Contributions

S.T.F.T. contributed to library preparation, sequencing, variant interpretation, statistical analysis, conception of the article and drafted the manuscript. C.K.P.L. contributed to variant interpretation and approved the final version. E.S.H.L. contributed to data acquisition, statistical analysis and approved the final version. R.C.W.M., B.F., C.K.O., Y.F., E.C., A.P.S.K., W.‐Y.S. and J.C.N.C. contributed to conception of the article and approved the final version. A.O.Y.L. contributed to conception of the article, supervision and revision of the manuscript, and approved the final version. A.O.Y.L. is the guarantor of this work, has full access to all the data in the study and takes responsibility for the integrity of the data and the accuracy of the data analysis.

## Conflicts of Interest

J.C.N.C. has received research grants and/or honoraria for consultancy and/or giving lectures from AstraZeneca, Bayer, Boehringer Ingelheim, Celltrion, Eli‐Lilly, Hua Medicine, Lee Powder, Merck Serono, Merck Sharp & Dohme, Pfizer, Servier, Sanofi and Viatris, and holds patents for using biomakers to predict risks of diabetes and its complications. R.C.W.M. has received research grants for clinical trials from AstraZeneca, Bayer, MSD, Novo Nordisk, Sanofi, Tricida Inc. and honoraria for consultancy or lectures from AstraZeneca, Bayer and Boehringer Ingelheim, all used to support diabetes research at the Chinese University of Hong Kong. J.C.N.C., R.C.W.M. and C.L. are co‐founders of GemVCare, a technology start‐up initiated with support from the Hong Kong Government Innovation and Technology Commission and its Technology Start‐up Support Scheme for Universities (TSSSU). A.P.S.K. has received honorarium for consultancy or giving lectures from Abbott, Astra Zeneca, Bayer, Boehringer Ingelheim, Eli‐Lilly, Kyowa Kirin, Merck Serono, Nestle, Novo‐Nordisk, Pfizer and Sanofi. No potential conflicts of interest relevant to this article were reported. A.O.Y.L. has received research grants for clinical trials from Amgen, AstraZeneca, Bayer, Boehringer Ingelheim, Merck Sharp & Dohme, Roche, and received honoraria for consultancy and/or giving lectures from Eli Lilly.

## Peer Review

The peer review history for this article is available at https://www.webofscience.com/api/gateway/wos/peer-review/10.1002/dmrr.70087.

## Supporting information


Supporting Information S1

## Data Availability

The authors have nothing to report.
