# Supplementary material for: Development of a Chinese‐Specific Clinical Model to Predict Maturity‐Onset Diabetes of the Young
Source: Diabetes Metab Res Rev. 2025 Sep 18;41(6):e70087. doi: 10.1002/dmrr.70087 (PMC12445802; doi:10.1002/dmrr.70087)
Supplement: Supplementary file 1 — Supporting Information S1 [file DMRR-41-e70087-s001.docx]

**Supporting information**

**Supplementary Method 1:** Variant interpretation

**Supplementary Method 2:** Alternative variable selection methods and sensitivity analysis

**Table S1**: List of monogenic diabetes genes included in the sequencing panel

**Table S2**: Proportion of missing data for baseline clinical characteristics and prevalent outcomes in the Hong Kong Diabetes Register (HKDR) cohort (training dataset) and the Precision Medicine to Redefine Insulin Secretion and Monogenic Diabetes (PRISM) study (validation dataset)

**Table S3**: Baseline clinical characteristics and prevalent outcomes in Chinese with MODY or without MODY in the HKDR cohort (training dataset)

**Table S4:** Baseline clinical characteristics and prevalent outcomes in Chinese with MODY subtypes in the HKDR cohort (training dataset)

**Table S5:** Baseline clinical characteristics and prevalent outcomes in Chinese with MODY in the HKDR cohort (training dataset) and the PRISM study (validation dataset)

**Table S6**: Bootstrap-derived estimates for the MODY prediction model

**Table S7:** Sensitivity analysis comparing the nine-variable model and reduced models

**Figure S1:** Development of prediction models by logistic regression and random forest.

**Figure S2:** Distribution of MODY-associated pathogenic variants identified in the HKDR and PRISM cohorts.

**Figure S3:** Examples of trees in random forest model.

**Figure S4.** ROC curve of the Exeter MODY probability calculator in validation dataset.

**Supplementary Method 1:** Variant interpretation

After next-generation sequencing and subsequent bioinformatic analysis, variants including single-nucleotide changes, insertions and deletions up to size of 100 bp were recorded and interpreted according to the guidelines developed by the American College of Medical Genetics and Genomics (ACMG) and the Association for Molecular Pathology (AMP) and the College of American Pathologists.^1^ The classification system evaluates pathogenicity of a variant in an evidence-based framework, integrating evidence of population data, predictive data, computational data, segregation data, functional data, *de novo* data, allelic data as well as phenotypic data from patients. The evidences of a variant were combined to determine its pathogenicity by following the scoring rules with posterior probabilities estimated by Bayesian approach.^2-4^ In addition, we adopted gene-specific specifications from the ClinGen Monogenic Diabetes Expert Panel (MDEP) for interpretation of variants in *GCK*, *HNF1A* and *HNF4A* genes.^5-7^

Variants were classified as “pathogenic” (P), “likely pathogenic” (LP), “variants of uncertain significance” (VUS), “likely benign” and “benign” based on the rules of combined scored evidence criteria from the ACMG/AMP guidelines.^1^ In this study, patients were considered to have MODY if they were heterozygous or homozygous carriers of P/LP variants in *GCK*, *HNF1A*, *HNF1B* and/or *HNF4A* gene.

References

1. Richards S, Aziz N, Bale S, et al. Standards and guidelines for the interpretation of sequence variants: a joint consensus recommendation of the American College of Medical Genetics and Genomics and the Association for Molecular Pathology. *Genet Med* 2015;17(5):405-424. https://doi.org/10.1038/gim.2015.30
2. Ellard S, Baple EL, Callaway A, et al. ACGS best practice guidelines for variant classification in rare disease 2020. Association for Clinical Genomic *Science.* Updated 2020. Accessed 11 August 2021. https://www.acgs.uk.com/media/11631/uk-practice-guidelines-for-variant-classification-v4-01-2020.pdf
3. Tavtigian SV, Harrison SM, Boucher KM, Biesecker LG. Fitting a naturally scaled point system to the ACMG/AMP variant classification guidelines. *Hum Mutat* 2020;41(10):1734-1737. https://doi.org/10.1002/humu.24088
4. Tavtigian SV, Greenblatt MS, Harrison SM, et al. Modeling the ACMG/AMP variant classification guidelines as a Bayesian classification framework. *Genet Med* 2018;20(9):1054-1060. https://doi.org/10.1038/gim.2017.210
5. Monogenic Diabetes Variant Curation Expert Panel. ClinGen Monogenic Diabetes Expert Panel Specifications to the ACMG/AMP Variant Interpretation Guidelines for HNF1A Version 2.1.0. 8 November 2023. Accessed 24 February 2024. https://cspec.genome.network/cspec/ui/svi/doc/GN017?version=2.1.0
6. Monogenic Diabetes Variant Curation Expert Panel. ClinGen Monogenic Diabetes Expert Panel Specifications to the ACMG/AMP Variant Interpretation Guidelines for GCK Version 1.3.0. 8 November 2023. Accessed 24 February 2024. https://cspec.genome.network/cspec/ui/svi/doc/GN086?version=1.3.0
7. Monogenic Diabetes Variant Curation Expert Panel. ClinGen Monogenic Diabetes Expert Panel Specifications to the ACMG/AMP Variant Interpretation Guidelines for HNF4A Version 2.0.0. 10 November 2023. Accessed 24 February 2024. https://cspec.genome.network/cspec/ui/svi/doc/GN085?version=2.0.0

**Supplementary Method 2:** Alternative variable selection methods and sensitivity analysis

In studies with limited sample sizes and few outcome events, careful variable selection is critical to minimize overfitting and ensure model stability. We evaluated alternative variable selection approaches for an optimal prediction model for MODY. Models were compared based on performance metrics such as sensitivity and specificity in the external validation cohort.

(1) Univariate analysis

Clinical variables were initially selected based on statistically significant differences between individuals with and without MODY (sex, current age, age at diagnosis, BMI, waist circumference, systolic blood pressure, HDL-cholesterol, LDL-cholesterol, triglyceride and fasting C-peptide values). Variance inflation factor (VIF) analysis was then performed to assess multicollinearity among the variables. Only variables with a VIF under 5 were retained in the logistic model. From the analysis, waist circumference and BMI were correlated with VIF >5. Therefore, waist circumference was excluded from the logistic regression model to reduce redundancy and increase model stability.

(2) Recursive feature elimination (RFE)

RFE was applied to the initial set of 20 clinical variables related to metabolic profile and medication use, for both logistic regression and random forest models. This method selected variables by recursively removing the least important features based on model-specific importance weights. However, RFE did not eliminate any variables and the models did not outperform the nine-variable logistic model derived from univariate analysis.

(3) Stepwise backward elimination

Stepwise backward elimination was applied to the logistic regression model. It iteratively removed the least statistically significant variable at each step, until all remaining variables were statistically significant. The final model obtained from backward elimination retained a similar set of variables (current age, BMI, systolic blood pressure, HDL-cholesterol, LDL-cholesterol and fasting C-peptide values) as the nine-variable model derived from univariate analysis, and did not improve predictive performance in the validation dataset. This method is not applicable to the random forest method, as it does not rely on p-values for feature significance.

(4) Sensitivity analysis

To further evaluate the contribution of multiple clinical variables on model performance, we performed sensitivity analysis by comparing the full nine-variables logistic model to two reduced models:

(a) A model including only fasting C-peptide and BMI, selected for their strong and consistent associations with MODY across both generalized linear model (GLM) and bootstrap analyses, as well as their established clinical relevance in distinguishing MODY and type 2 diabetes.

(b) A model including only variables that demonstrated statistically significance in the full logistic regression model, that are current age, BMI, systolic blood pressure, HDL-cholesterol, LDL-cholesterol and fasting C-peptide values (Table 1).

The predictive performance of these reduced models were assessed using the same external validation dataset and probability cut-off ≥50% (Table S7). Theses comparisons support the use of a broader set of clinically relevant variables for prediction model construction, even if not all are statistically significant individually. As a result, we retained the nine variables identified through univariate analysis for model development.

**Table S1**: List of monogenic diabetes genes included in the sequencing panel

| **Gene**^†^ | **Mode of inheritance** | **Phenotypes** | **GenBank Reference Sequence** |
| --- | --- | --- | --- |
| *ABCC8* | AD | MODY | NM_000352 |
|  | AR | CHI, NDM |  |
| *AKT2* | AD | Lipodystrophy, IR | NM_001626 |
| *APPL1* | AD | MODY | NM_012096 |
| *CEL*^‡^ | AD | MODY, Syndrome | NM_001807 |
| *CISD2* | AR | Syndrome | NM_001008388 |
| *DCAF17* | AR | Syndrome | NM_025000 |
| *DNAJC3* | AR | Syndrome | NM_006260 |
| *DYRK1B* | AD | Syndrome | NM_004714 |
| *GATA4*^‡^ | AD | NDM, Syndrome | NM_002052 |
| *GATA6* | AD | NDM, Syndrome | NM_005257 |
| *GCK* | AD | MODY | NM_000162 |
|  | AR | NDM |  |
| *HNF1A* | AD | MODY | NM_000545 |
| *HNF1B*^§^ | AD | NDM, MODY, Syndrome | NM_000458 |
| *HNF4A* | AD | MODY | NM_175914 |
| *INS* | AD | NDM, MODY | NM_000207 |
|  | AR | NDM |  |
| *INSR* | AD, AR | IR, NDM, Syndrome | NM_000208 |
| *KCNJ11*^‡^ | AD | CHI, NDM, MODY | NM_000525 |
| *LMNA* | AD | Lipodystrophy, IR, Syndrome | NM_170707 |
| *NEUROD1*^‡^ | AD | MODY | NM_002500 |
|  | AR | NDM |  |
| *PAX6* | AD | Syndrome | NM_001368894 |
| *PCBD1* | AR | Syndrome | NM_000281 |
| *PDX1*^‡^ | AD | MODY | NM_000209 |
|  | AR | NDM |  |
| *PIK3R1* | AD | Syndrome | NM_181523 |
| *PLIN1* | AD | Lipodystrophy, IR, Syndrome | NM_002666 |
| *POLD1*^‡^ | AD | Lipodystrophy, IR, Syndrome | NM_002691 |
| *PPARG* | AD | Lipodystrophy, IR, Syndrome | NM_015869 |
| *PPP1R15B* | AR | Syndrome | NM_032833 |
| *RFX6* | AD | MODY | NM_173560 |
|  | AR | NDM, Syndrome |  |
| *SLC29A3* | AR | NDM, Syndrome | NM_018344 |
| *TRMT10A* | AR | Syndrome | NM_001134665 |
| *WFS1* | AD, AR | NDM, Syndrome | NM_006005 |
| *ZBTB20* | AD | Syndrome | NM_001348800 |
| *ZFP57* | AR | NDM, MODY, Syndrome | NM_001109809 |

Abbreviations: AD, autosomal dominant; AR, autosomal recessive; CHI, congenital hyperinsulinism; IR, insulin resistance; MODY, maturity-onset diabetes of the young; NDM, neonatal diabetes mellitus.

^†^ The sequencing regions covered exons and flanking regions located within 25 base pairs (bp) upstream and downstream of each exon.

^‡^ The coding and flanking regions of the genes *CEL* (87.3%), *GATA4* (95.2%), *KCNJ11* (90.1%), *NEUROD1* (96.3%), *PDX1* (94.0%) and *POLD1* (96.4%) were partially covered due to technical limitations of designing target-specific primers for certain sequences.

^§^ Copy numbers of *HNF1B* region were analyzed from sequencing reads using a copy number variation (CNV) detection algorithm CNVPanelizer. CNV identified were further validated by multiplex ligation-dependent probe amplification (MLPA) (SALSA MLPA P241 MODY kit, MRC Holland, Amsterdam, Netherlands).

**Table S2**: Proportion of missing data for baseline clinical characteristics and prevalent outcomes in the Hong Kong Diabetes Register (HKDR) cohort (training dataset) and the Precision Medicine to Redefine Insulin Secretion and Monogenic Diabetes (PRISM) study (validation dataset)

|  | **HKDR** | **PRISM** |
| --- | --- | --- |
| Total number of patients | 1,021 | 822 |
| Current age | 0 (0.0%) | 0 (0.0%) |
| Sex | 0 (0.0%) | 0 (0.0%) |
| Age at diabetes diagnosis | 0 (0.0%) | 0 (0.0%) |
| BMI^†^ | 4 (0.4%) | 0 (0.0%) |
| Waist circumference (men)^†^ | 1 (0.2%) | 5 (1.1%) |
| Waist circumference (women)^†^ | 3 (0.5%) | 2 (0.6%) |
| Systolic blood pressure^†^ | 2 (0.2%) | 0 (0.0%) |
| HDL-cholesterol^†^ | 10 (1.0%) | 10 (1.2%) |
| LDL-cholesterol^†^ | 59 (5.8%) | 39 (4.7%) |
| Triglyceride^†^ | 3 (0.3%) | 6 (0.7%) |
| Fasting C-peptide | 0 (0.0%) | 0 (0.0%) |

Values are presented as n (%), where n represents the number of individuals with missing data, and % represents the proportion of missing data relative to the total sample size of each cohort.

Abbreviations: BMI, body mass index; HDL, high-density lipoprotein; LDL, low-density lipoprotein.

^†^ Missing data were imputed by the median value in the corresponding dataset.

**Table S3**: Baseline clinical characteristics and prevalent outcomes in Chinese with MODY or without MODY in the HKDR cohort (training dataset)

|  | **Without MODY** | **MODY** | **p-value** |
| --- | --- | --- | --- |
| Number (%) | 1002 (98.1%) | 19 (1.9%) |  |
| **Demographics and lifestyle** | | | |
| Current age, years | 39.9 (35.2-47.8) | 34.6 (20.6-39.8) | **0.003**** |
| Men, n (%) | 452 (45.1%) | 6 (31.6%) | 0.257 |
| Current or ex-smokers, n (%) | 267 (26.8%) | 2 (10.5%) | 0.124 |
| **Metabolic profile** | | | |
| Age at diabetes diagnosis, years | 35.0 (30.0-38.0) | 31.0 (19.0-35.0) | **0.017*** |
| Proportion diagnosed diabetes at age <25 years, n (%) | 97 (9.7%) | 5 (26.3%) | **0.034*** |
| Time from diabetes diagnosis, years | 6.0 (1.0-13.0) | 3.0 (1.0-6.0) | 0.097 |
| Paternal history of diabetes, n (%) | 223 (23.5%) | 3 (17.6%) | 0.775 |
| Maternal history of diabetes, n (%) | 340 (35.8%) | 8 (44.4%) | 0.464 |
| Family history of diabetes, n (%) | 563 (56.2%) | 12 (63.2%) | 0.644 |
| BMI, kg/m^2^ | 25.5 (22.7-28.8) | 21.6 (20.1-23.8) | **<0.001***** |
| Waist circumference (men), cm | 89.0 (81.0-96.0) | 71.8 (71.0-93.0) | **0.047*** |
| Waist circumference (women), cm | 83.0 (76.0-90.0) | 70.0 (65.3-77.5) | **<0.001***** |
| Systolic blood pressure, mmHg | 123.8 (114.0-136.0) | 116.0 (109.5-123.5) | **0.038*** |
| Diastolic blood pressure, mmHg | 74.0 (67.5-81.5) | 71.0 (63.5-80.0) | 0.184 |
| HbA1c, mmol/mol | 58.5 (47.5-73.8) | 49.7 (45.4-65.0) | 0.116 |
| HbA1c, % | 7.5 (6.5-8.9) | 6.7 (6.3-8.1) | 0.116 |
| Fasting plasma glucose, mmol/L | 8.0 (6.4-10.7) | 7.3 (6.5-9.9) | 0.241 |
| HDL-cholesterol, mmol/L | 1.24 (1.04-1.50) | 1.60 (1.08-1.87) | **0.007**** |
| LDL-cholesterol, mmol/L | 2.83 (2.30-3.50) | 2.40 (1.97-2.90) | **0.038*** |
| Triglyceride, mmol/L | 1.38 (0.90-2.17) | 0.88 (0.70-1.28) | **0.005**** |
| Estimated GFR, ml/min/1.73m^2^ | 103.3 (89.0-114.0) | 108.4 (86.5-121.2) | 0.382 |
| Urine ACR, mg/mmol | 1.63 (0.67-6.93) | 0.70 (0.50-4.50) | 0.144 |
| Fasting C-peptide, pmol/L^†^ | 265.5 (50.0-625.2) | 50.0 (50.0-223.1) | **0.005**** |
| Positive anti-GAD autoantibodies, n (%)^‡^ | 72 (7.2%) | 2 (10.5%) | 0.642 |
| **Diabetes-related comorbidities and complications** | | | |
| Overweight or obesity, n (%) | 717 (71.8%) | 5 (27.8%) | **<0.001***** |
| Hypertension, n (%) | 561 (56.0%) | 6 (31.6%) | **0.038*** |
| Dyslipidemia, n (%) | 695 (72.5%) | 7 (36.8%) | **0.001**** |
| Albuminuria, n (%) | 356 (36.7%) | 4 (22.2%) | 0.230 |
| CKD, n (%) | 67 (6.7%) | 2 (10.5%) | 0.372 |
| ESKD, n (%) | 9 (0.9%) | 0 (0.0%) | 1.000 |
| Diabetic sensory neuropathy, n (%) | 128 (12.8%) | 1 (5.3%) | 0.496 |
| Diabetic retinopathy, n (%) | 237 (24.6%) | 3 (15.8%) | 0.589 |
| Coronary heart disease, n (%) | 34 (3.4%) | 0 (0.0%) | 1.000 |
| Peripheral artery disease, n (%) | 22 (2.2%) | 0 (0.0%) | 1.000 |
| Stroke, n (%) | 13 (1.3%) | 0 (0.0%) | 1.000 |
| **Medications** | | | |
| Oral glucose-lowering drugs, n (%) | 634 (65.9%) | 10 (52.6%) | 0.329 |
| Insulin, n (%) | 216 (22.5%) | 2 (10.5%) | 0.275 |
| Lipid-lowering drugs, n (%) | 151 (15.1%) | 2 (10.5%) | 0.755 |
| Blood pressuring lowering drugs, n (%) | 294 (29.3%) | 3 (15.8%) | 0.219 |
| Renin-angiotensin system blockers, n (%) | 183 (19.0%) | 1 (5.3%) | 0.229 |

Comparison conducted by chi-squared test or Fisher’s Exact test for comparison of categorical variables and Wilcoxon rank-sum test for comparison of nonparametric data when applicable. Results were presented as median (interquartile range [IQR]) or number (%) as appropriate.

Abbreviations: ACR, albumin-to-creatinine ratio; BMI, body mass index; CKD, chronic kidney disease; ESKD, end-stage kidney disease; GAD, glutamic acid decarboxylase; GFR, glomerular filtration rate; HDL, high-density lipoprotein; LDL, low-density lipoprotein.

Albuminuria: urine ACR >3.0 mg/mmol; CKD: estimated GFR <60 ml/min/1.73m^2^; Dyslipidemia: LDL-cholesterol ≥2.6 mmol/L or use of lipid regulating drug; ESKD: estimated GFR <15 ml/min/1.73m^2^, dialysis or previous renal transplant; Hypertension: systolic blood pressure ≥130 mmHg, diastolic blood pressure ≥80 mmHg or use of blood pressure lowering drugs; Overweight or obesity: BMI equal or greater than 23.0 kg/m^2^.

*p <0.05, **p <0.01, ***p <0.001.

^†^ When C-peptide level was lower than the kit detection limits (<95.8 or <97.9 pmol/L), the level was classified as undetectable; a median value of 50.0 pmol/L was assumed for patients with undetectable C-peptide.

^‡^ Anti-GAD autoantibodies were detected at titre >5.0 U/mL.

**Table S4**: Baseline clinical characteristics and prevalent outcomes in Chinese with MODY subtypes in the HKDR cohort (training dataset)

| Subtypes | ***GCK*-MODY** | **Transcription factor-MODY** | **p-value** |
| --- | --- | --- | --- |
| Number (%) | 6 (31.6%) | 13 (68.4%) |  |
| **Demographics and lifestyle** | | | |
| Current age, years | 36.6 (30.4-40.1) | 33.9 (19.7-41.2) | 0.579 |
| Men, n (%) | 1 (16.7%) | 5 (38.5%) | 0.605 |
| Current or ex-smokers, n (%) | 1 (16.7%) | 1 (7.7%) | 1.000 |
| **Metabolic profile** | | | |
| Age at diabetes diagnosis, years | 33.5 (29.5-38.5) | 31.0 (17.0-33.0) | 0.127 |
| Proportion diagnosed diabetes at age <25 years, n (%) | 0 (0.0%) | 5 (38.5%) | 0.128 |
| Time from diabetes diagnosis, years | 1.0 (0.8-3.5) | 5.0 (0.5-9.5) | 0.179 |
| Paternal history of diabetes, n (%) | 1 (16.7%) | 2 (18.2%) | 1.000 |
| Maternal history of diabetes, n (%) | 2 (33.3%) | 6 (50.0%) | 0.638 |
| Family history of diabetes, n (%) | 3 (50.0%) | 9 (69.2%) | 0.617 |
| BMI, kg/m^2^ | 22.0 (19.8-25.9) | 21.4 (20.0-23.3) | 0.750 |
| Waist circumference (men), cm | 91.0 (91.0-91.0) | 71.5 (71.0-85.5) | 0.667 |
| Waist circumference (women), cm | 73.0 (66.3-86.0) | 69.5 (62.0-74.3) | 0.284 |
| Systolic blood pressure, mmHg | 119.0 (106.8-122.0) | 114.5 (109.8-123.8) | 1.000 |
| Diastolic blood pressure, mmHg | 78.0 (67.3-83.5) | 67.0 (61.5-74.3) | 0.210 |
| HbA1c, mmol/mol | 47.5 (45.4-53.0) | 51.9 (46.4-69.4) | 0.282 |
| HbA1c, % | 6.5 (6.3-7.0) | 6.9 (6.4-8.5) | 0.282 |
| Fasting plasma glucose, mmol/L | 7.0 (6.6-8.5) | 7.4 (5.7-10.2) | 0.701 |
| HDL-cholesterol, mmol/L | 1.60 (1.03-1.77) | 1.60 (1.14-1.90) | 0.521 |
| LDL-cholesterol, mmol/L | 2.70 (2.29-3.35) | 2.40 (1.77-2.71) | 0.179 |
| Triglyceride, mmol/L | 1.16 (0.70-2.42) | 0.85 (0.66-1.14) | 0.282 |
| Estimated GFR, ml/min/1.73m^2^ | 104.5 (87.8-122.3) | 110.1 (75.4-123.1) | 1.000 |
| Urine ACR, mg/mmol | 0.7 (0.5-1.3) | 1.1 (0.5-16.7) | 0.553 |
| Fasting C-peptide, pmol/L^†^ | 50.0 (50.0-67.8) | 187.8 (50.0-266.4) | 0.058 |
| Proportion with undetectable fasting C-peptide, n (%)^†^ | 5 (83.3%) | 5 (38.5%) | 0.141 |
| Positive anti-GAD autoantibodies, n (%)^‡^ | 1 (16.7%) | 1 (7.7%) | 1.000 |
| **Diabetes-related comorbidities and complications** | | | |
| Overweight or obesity, n (%) | 2 (33.3%) | 3 (25.0%) | 1.000 |
| Hypertension, n (%) | 3 (50.0%) | 3 (23.1%) | 0.320 |
| Dyslipidemia, n (%) | 3 (50.0%) | 4 (30.8%) | 0.617 |
| Albuminuria, n (%) | 0 (0.0%) | 4 (33.3%) | 0.245 |
| CKD, n (%) | 0 (0.0%) | 2 (15.4%) | 1.000 |
| ESKD, n (%) | 0 (0.0%) | 0 (0.0%) | - |
| Diabetic sensory neuropathy, n (%) | 0 (0.0%) | 1 (7.7%) | 1.000 |
| Diabetic retinopathy, n (%) | 0 (0.0%) | 3 (23.1%) | 0.517 |
| Coronary heart disease, n (%) | 0 (0.0%) | 0 (0.0%) | - |
| Peripheral artery disease, n (%) | 0 (0.0%) | 0 (0.0%) | - |
| Stroke, n (%) | 0 (0.0%) | 0 (0.0%) | - |
| **Medications** | | | |
| Oral glucose-lowering drugs, n (%) | 1 (16.7%) | 9 (69.2%) | 0.057 |
| Insulin, n (%) | 1 (16.7%) | 1 (7.7%) | 1.000 |
| Lipid-lowering drugs, n (%) | 0 (0.0%) | 2 (15.4%) | 1.000 |
| Blood pressuring lowering drugs, n (%) | 0 (0.0%) | 3 (23.1%) | 0.517 |
| Renin-angiotensin system blockers, n (%) | 0 (0.0%) | 1 (7.7%) | 1.000 |

Comparison conducted by chi-squared test or Fisher’s Exact test for comparison of categorical variables and Wilcoxon rank-sum test for comparison of nonparametric data when applicable. Results were presented as median (interquartile range [IQR]) or number (%) as appropriate.

Abbreviations: ACR, albumin-to-creatinine ratio; BMI, body mass index; CKD, chronic kidney disease; ESKD, end-stage kidney disease; GAD, glutamic acid decarboxylase; GFR, glomerular filtration rate; HDL, high-density lipoprotein; LDL, low-density lipoprotein.

Albuminuria: urine ACR >3.0 mg/mmol; CKD: estimated GFR <60 ml/min/1.73m^2^; Dyslipidemia: LDL-cholesterol ≥2.6 mmol/L or use of lipid regulating drug; ESKD: estimated GFR <15 ml/min/1.73m^2^, dialysis or previous renal transplant; Hypertension: systolic blood pressure ≥130 mmHg, diastolic blood pressure ≥80 mmHg or use of blood pressure lowering drugs; Overweight or obesity: BMI equal or greater than 23.0 kg/m^2^.

*p <0.05, **p <0.01, ***p <0.001.

^†^ When C-peptide level was lower than the kit detection limits (<95.8 or <97.9 pmol/L), the level was classified as undetectable; a median value of 50.0 pmol/L was assumed for patients with undetectable C-peptide.

^‡^ Anti-GAD autoantibodies were detected at titre >5.0 U/mL.

**Table S5**: Baseline clinical characteristics and prevalent outcomes in Chinese with MODY in the HKDR cohort (training dataset) and the PRISM study (validation dataset)

| Cohorts | **HKDR** | **PRISM** | **p-value** |
| --- | --- | --- | --- |
| Number (%) | 19 (1.9%) | 11 (1.3%) |  |
| **Demographics and lifestyle** | | | |
| Current age, years | 34.6 (20.6-39.8) | 42.5 (31.5-46.2) | 0.094 |
| Men, n (%) | 6 (31.6%) | 4 (36.4%) | 1.000 |
| Current or ex-smokers, n (%) | 2 (10.5%) | 0 (0.0%) | 0.520 |
| **Metabolic profile** | | | |
| Age at diabetes diagnosis, years | 31.0 (19.0-35.0) | 29.0 (25.0-35.0) | 0.641 |
| Proportion diagnosed diabetes at age <25 years, n (%) | 5 (26.3%) | 2 (18.2%) | 1.000 |
| Time from diabetes diagnosis, years | 3.0 (1.0-6.0) | 10.0 (6.0-17.0) | **0.003**** |
| Paternal history of diabetes, n (%) | 3 (17.6%) | 4 (36.4%) | 0.381 |
| Maternal history of diabetes, n (%) | 8 (44.4%) | 5 (45.5%) | 1.000 |
| Family history of diabetes, n (%) | 12 (63.2%) | 8 (72.7%) | 0.702 |
| BMI, kg/m^2^ | 21.6 (20.1-23.8) | 22.3 (19.0-23.5) | 0.947 |
| Waist circumference (men), cm | 71.8 (71.0-93.0) | 82.5 (71.1-94.3) | 0.914 |
| Waist circumference (women), cm | 70.0 (65.3-77.5) | 79.0 (74.0-82.0) | 0.067 |
| Systolic blood pressure, mmHg | 116.0 (109.5-123.5) | 122.0 (113.0-143.0) | 0.268 |
| Diastolic blood pressure, mmHg | 71.0 (63.5-80.0) | 75.0 (66.0-82.0) | 0.395 |
| HbA1c, mmol/mol | 49.7 (45.4-65.0) | 56.3 (48.6-61.7) | 0.350 |
| HbA1c, % | 6.7 (6.3-8.1) | 7.3 (6.6-7.8) | 0.350 |
| Fasting plasma glucose, mmol/L | 7.3 (6.5-9.9) | 6.9 (5.2-7.2) | 0.134 |
| HDL-cholesterol, mmol/L | 1.60 (1.08-1.87) | 1.50 (1.20-2.00) | 0.611 |
| LDL-cholesterol, mmol/L | 2.40 (1.97-2.90) | 2.30 (2.20-2.60) | 0.497 |
| Triglyceride, mmol/L | 0.88 (0.70-1.28) | 0.60 (0.60-1.40) | 0.350 |
| Estimated GFR, ml/min/1.73m^2^ | 108.4 (86.5-121.2) | 105.5 (93.0-115.0) | 0.703 |
| Urine ACR, mg/mmol | 0.67 (0.51-4.50) | 0.80 (0.40-2.42) | 0.877 |
| Fasting C-peptide, pmol/L^†^ | 50.0 (50.0-223.1) | 268.4 (101.8-578.1) | **0.023*** |
| Proportion with undetectable fasting C-peptide, n (%)^†^ | 10 (52.6%) | 2 (18.2%) | 0.121 |
| Positive anti-GAD autoantibodies, n (%)^‡^ | 2 (10.5%) | 0 (0.0%) | 0.520 |
| **Diabetes-related comorbidities and complications** | | | |
| Overweight or obesity, n (%) | 5 (27.8%) | 3 (27.3%) | 1.000 |
| Hypertension, n (%) | 6 (31.6%) | 4 (36.4%) | 1.000 |
| Dyslipidemia, n (%) | 7 (36.8%) | 8 (72.7%) | 0.128 |
| Albuminuria, n (%) | 4 (22.2%) | 2 (18.2%) | 1.000 |
| CKD, n (%) | 2 (10.5%) | 1 (9.1%) | 1.000 |
| ESKD, n (%) | 0 (0.0%) | 0 (0.0%) | - |
| Diabetic sensory neuropathy, n (%) | 1 (5.3%) | 0 (0.0%) | 1.000 |
| Diabetic retinopathy, n (%) | 3 (15.8%) | 3 (27.3%) | 0.641 |
| Coronary heart disease, n (%) | 0 (0.0%) | 0 (0.0%) | - |
| Peripheral artery disease, n (%) | 0 (0.0%) | 0 (0.0%) | - |
| Stroke, n (%) | 0 (0.0%) | 0 (0.0%) | - |
| **Medications** | | | |
| Oral glucose-lowering drugs, n (%) | 10 (52.6%) | 9 (81.8%) | 0.140 |
| Insulin, n (%) | 2 (10.5%) | 3 (27.3%) | 0.327 |
| Lipid-lowering drugs, n (%) | 2 (10.5%) | 6 (54.5%) | **0.028*** |
| Blood pressuring lowering drugs, n (%) | 3 (15.8%) | 4 (36.4%) | 0.372 |
| Renin-angiotensin system blockers, n (%) | 1 (5.3%) | 1 (12.5%) | 0.513 |

Comparison conducted by chi-squared test or Fisher’s Exact test for comparison of categorical variables and Wilcoxon rank-sum test for comparison of nonparametric data when applicable. Results were presented as median (interquartile range [IQR]) or number (%) as appropriate.

Abbreviations: ACR, albumin-to-creatinine ratio; BMI, body mass index; CKD, chronic kidney disease; ESKD, end-stage kidney disease; GAD, glutamic acid decarboxylase; GFR, glomerular filtration rate; HDL, high-density lipoprotein; LDL, low-density lipoprotein.

Albuminuria: urine ACR >3.0 mg/mmol; CKD: estimated GFR <60 ml/min/1.73m^2^; Dyslipidemia: LDL-cholesterol ≥2.6 mmol/L or use of lipid regulating drug; ESKD: estimated GFR <15 ml/min/1.73m^2^, dialysis or previous renal transplant; Hypertension: systolic blood pressure ≥130 mmHg, diastolic blood pressure ≥80 mmHg or use of blood pressure lowering drugs; Overweight or obesity: BMI equal or greater than 23.0 kg/m^2^.

*p <0.05, **p <0.01, ***p <0.001.

^†^ When C-peptide level was lower than the kit detection limits (<95.8 or <97.9 pmol/L), the level was classified as undetectable; a median value of 50.0 pmol/L was assumed for patients with undetectable C-peptide.

^‡^ Anti-GAD autoantibodies were detected at titre >5.0 U/mL.**Table S6**: Bootstrap-derived estimates for the MODY prediction model

| **Variable** | **Empirical  95% CI OR** | **Empirical SE** | **Empirical  z score** | **Empirical  p-value** |
| --- | --- | --- | --- | --- |
| Sex (male=0, female =1) | 0.423-27.809 | 0.98 | 0.57 | 0.571 |
| Current age (years) | 0.676-1.024 | 0.10 | -0.93 | 0.355 |
| Age at diagnosis (years) | 0.845-1.232 | 0.10 | -0.08 | 0.936 |
| BMI (kg/m^2^) | 0.543-0.974 | 0.15 | -1.54 | 0.124 |
| Systolic blood pressure (mmHg) | 0.951-1.097 | 0.03 | 0.57 | 0.570 |
| HDL-cholesterol (mmol/L) | 0.239-22.784 | 1.16 | 0.58 | 0.561 |
| LDL-cholesterol (mmol/L) | 0.170-1.422 | 0.53 | -0.99 | 0.323 |
| Fasting C-peptide (pmol/L) | 0.989-0.997 | 0.00 | -1.81 | 0.071 |
| Triglyceride (mmol/L) | 0.179-3.903 | 0.79 | -0.19 | 0.846 |

Empirical 95% confidence intervals (CIs), standard errors (SEs), z scores and p-values were obtained from bootstrap resampling with 10,000 iterations.

Abbreviations: CI, confidence interval; OR, odds ratio; SE, standard error.

**Table S7:** Sensitivity analysis comparing the nine-variable model and reduced models

| **Metrics** | **Full model**  **(Nine variables)**^†^ | **Reduced model (Two variables)^‡^** | **Reduced model**  **(Six variables)**^§^ |
| --- | --- | --- | --- |
| Area under the curve (AUC) | 0.813 | 0.820 | 0.816 |
| Sensitivity | 72.7% | 63.6% | 72.7% |
| Specificity | 92.4% | 90.4% | 91.9% |
| Positive predictive value (PPV) | 11.4% | 8.2% | 10.8% |
| False negative rate (FNR) | 27.3% | 36.4% | 27.3% |
| In population of 1000 people | | | |
| Number of tests needed | 85.2 | 103.4 | 90.0 |
| Number of positive cases identified/ missed | 9.7/3.6 | 8.5/4.9 | 9.7/3.6 |

Both models were evaluated using the same training dataset with Ridge-regularized logistic regression and class balancing at probability cut-off ≥50%.

^†^ Sex, current age, age at diagnosis, BMI, systolic blood pressure, HDL-cholesterol, LDL-cholesterol, triglyceride and fasting C-peptide values were included in the model.

^‡^ BMI and fasting C-peptide values were included in the model.

^§^ Current age, BMI, systolic blood pressure, HDL-cholesterol, LDL-cholesterol and fasting C-peptide values were included in the model.

**Figure S1.** Development of prediction models by logistic regression and random forest.

**Figure S2.** Distribution of MODY-associated pathogenic variants identified in the HKDR (a) and PRISM (b) cohorts.

**Figure S3.** Examples of trees in random forest model: The 1^st^ (a), 11^th^ (b), 21^st^ (c) and 31^st^ (d) tree among 1500 trees in model.

| a | b |
| --- | --- |
| 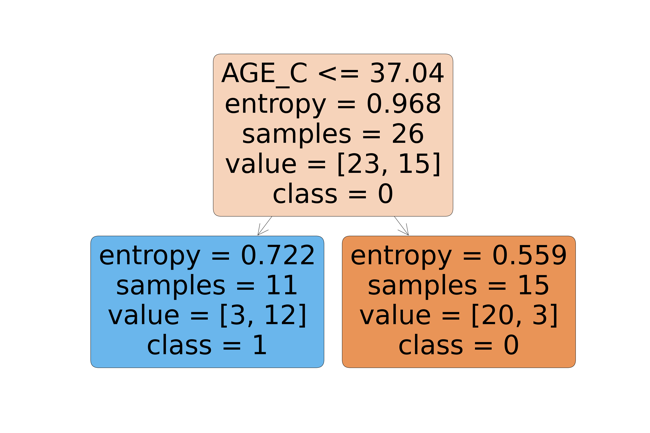 | 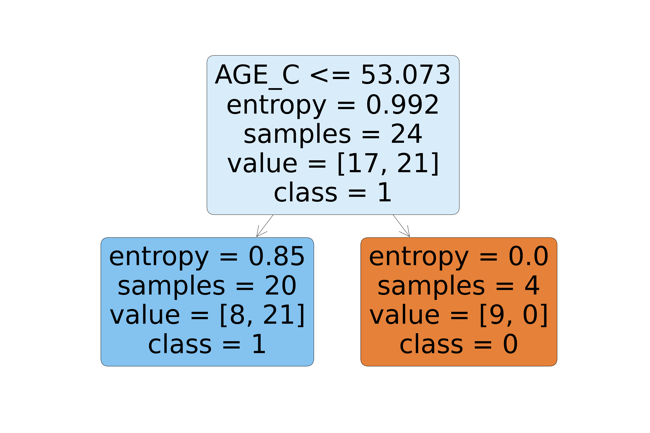 |
| c | d |
| 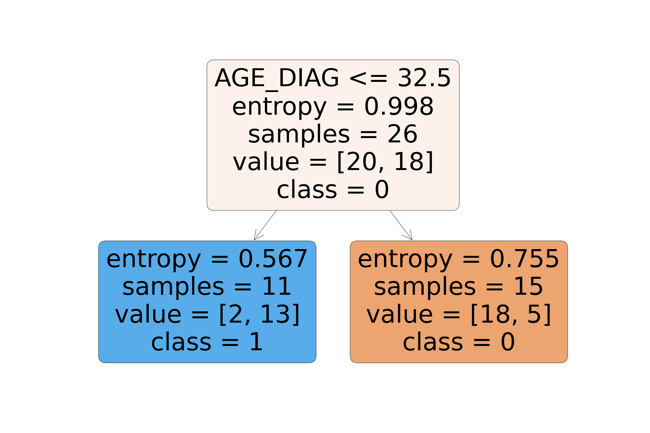 | 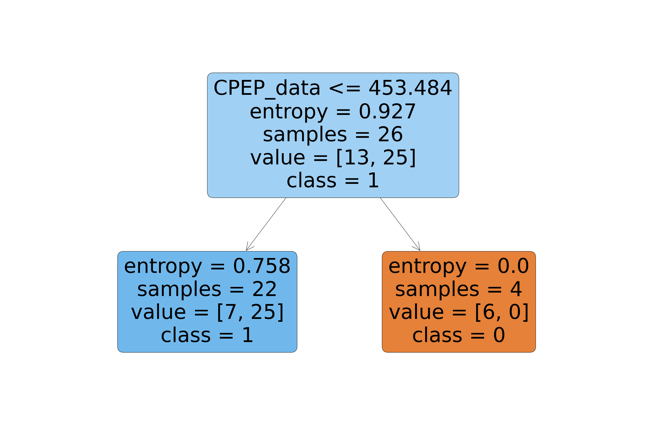 |

Abbreviations: AGE_C, current age; AGE_DIAG, age at diagnosis; CPEP_data; fasting C-peptide value.

**Figure S4.** ROC curve of the Exeter MODY probability calculator in validation dataset.
